# Managing for Interactions between Local and Global Stressors of Ecosystems

**DOI:** 10.1371/journal.pone.0065765

**Published:** 2013-06-12

**Authors:** Christopher J. Brown, Megan I. Saunders, Hugh P. Possingham, Anthony J. Richardson

**Affiliations:** 1 The Global Change Institute and the School of Biological Sciences, The University of Queensland, St Lucia, Queensland, Australia; 2 Australian Research Council, Centre for Excellence for Environmental Decisions, School of Biological Sciences, The University of Queensland, St Lucia, Queensland, Australia; 3 Climate Adaptation Flagship, Commonwealth Scientific and Industrial Research Organisation, Marine and Atmospheric Research, Dutton Park, Queensland, Australia; 4 Centre for Applications in Natural Resource Mathematics, School of Mathematics and Physics, The University of Queensland, St Lucia, Queensland, Australia; University of Gothenburg, Sweden

## Abstract

Global stressors, including climate change, are a major threat to ecosystems, but they cannot be halted by local actions. Ecosystem management is thus attempting to compensate for the impacts of global stressors by reducing local stressors, such as overfishing. This approach assumes that stressors interact additively or synergistically, whereby the combined effect of two stressors is at least the sum of their isolated effects. It is not clear, however, how management should proceed for antagonistic interactions among stressors, where multiple stressors do not have an additive or greater impact. Research to date has focussed on identifying synergisms among stressors, but antagonisms may be just as common. We examined the effectiveness of management when faced with different types of interactions in two systems – seagrass and fish communities – where the global stressor was climate change but the local stressors were different. When there were synergisms, mitigating local stressors delivered greater gains, whereas when there were antagonisms, management of local stressors was ineffective or even degraded ecosystems. These results suggest that reducing a local stressor can compensate for climate change impacts if there is a synergistic interaction. Conversely, if there is an antagonistic interaction, management of local stressors will have the greatest benefits in areas of refuge from climate change. A balanced research agenda, investigating both antagonistic and synergistic interaction types, is needed to inform management priorities.

## Introduction

Ensuring the persistence of critical habitats, dependent communities and ecological processes requires simultaneous management of multiple local and global stressors caused by human activities [1], [2]. A stressor is an environmental variable that negatively affects individual physiology or population performance when it is beyond its normal range of variation [3]. Generally, local stressors can be manipulated directly by management. Examples include improving water quality to halt declines of seagrass, coral reef and near-shore communities [4], maintaining riparian forest to buffer streams from run-off [5], and creating reserves to slow deforestation of tropical forests [6]. Increasingly, stressors with global causes are major drivers of ecosystem change. In particular, global climate change threatens habitats, ecological communities and ecological processes [7], [8]. For instance, extreme temperature events threaten the persistence of seagrass beds in the Mediterranean [9], drought and fire threaten fragmented forests [10], heat waves and ocean acidification threaten coral reef habitat and dependent fish communities [11], [12], and warming threatens numerous species with extinction [13]. Reducing global stressors requires collaboration among countries or regional management bodies, so they are not amenable to manipulation directly by management at a local scale. Therefore, management at a local scale can only act on impacts of global stressors indirectly, by reducing local stressors.

Stressors can have interactive effects on populations and ecosystems. If there is no interaction, the combined effect of two stresses is said to be additive, which is the sum of their effects in isolation. Interactions between stresses can be synergistic, where the combined effect of two stresses is greater than the additive expectation. Interactions may also be antagonistic, where the combined effect is less than the additive expectation. Stressors and their interactions can act at different levels of ecological organisation. A species may be subject to a synergistic interaction when the presence of one stressor reduces the physiological tolerance of individuals to additional stresses. For example, some corals may be hyper-sensitive to thermal bleaching if they are already physiologically stressed by poor water quality [14]. At a population level, if individuals tolerant of one stressor are sensitive to another, multiple stressors will tend to have a synergistic effect on mortality [3]. Antagonisms may occur when a population is made up of individuals that are either tolerant or sensitive to stress (co-variability), regardless of the stressor’s identity. For instance, if one stressor removes the most sensitive individuals, the remaining population will be tolerant of additional stressors [3]. More rarely, antagonisms can have mitigative effects on individuals. High sediment levels can reduce survival of coral colonies [15], but, sediments can be beneficial for corals at risk of bleaching, because reduced water clarity may reduce physiological light stress on corals [15]. At the community level, co-variability in stressor tolerance among species can also lead to antagonisms or synergisms, in a similar way that variability among individuals in a population can lead to interactive effects on populations [3].

We propose there are three prevailing views about interactions in the management of global stressors of ecosystems. The first is that synergisms are prevalent [16]. Synergisms are of concern, because future rates of ecosystem decline predicted on the basis of individual stressor effects will be underestimated if there are synergistic interactions between stressors [16], [17]. Synergisms will also cause more rapid declines in ecosystems than additive or antagonistic interactions. This view implies that management of local stressors can benefit ecosystems impacted by global stressors. A second view is that multiple stressors have cumulative impacts on ecosystems (e.g. [1], [18]). While useful for identifying the large-scale impacts of humans on ecosystems, such studies assume additive interactions and imply that management that addresses the largest stressor will have the greatest benefit [18], [19]. The final view is managing for ecological resilience [20]. This generally entails managing a local stressor to reduce the likelihood of ecological transitions to alternative degraded states, such as coral reefs to macro-algal dominated reefs, or desertification of grasslands [21], [22]. This may include reducing a local stressor, such as fishing, to improve recovery rates from, or resistance to, uncontrollable disturbances, such as hurricanes and climate change [23], [24].

None of these views addresses the prevalence of antagonistic interactions between stressors; they all assume that managing a local stressor improves the ecosystem. Antagonisms imply that local management actions cannot compensate for global stressors such as climate change impacts. Recent meta-analyses of experimental studies from marine, freshwater and terrestrial systems indicate that antagonisms are just as common as synergisms. At both population and community levels; antagonistic and synergistic interactions each made up approximately a third of all interactions [16], [17]. There is also evidence that individuals and species with lower metabolic rates tend to be more tolerant to multiple different kinds of physiological stress [25]. This implies co-tolerance in sensitivity to multiple stressors and suggests that antagonisms will be prevalent. There is concern that the focus on synergisms over antagonisms will result in ineffective management actions and wasted management effort [20], [26]. While this concern follows intuitively from empirical studies, there is a dearth of modelling studies for understanding the effectiveness of local management actions on outcomes for populations and communities.

Our approach is to build simple models to illustrate how interactions between climate and local stressors matter for the management of populations and communities. We use two case study models to illustrate interactions at both population and community levels. First, we use a population model of a seagrass bed to examine the expected number of years before a seagrass bed is degraded beyond recovery. The gain local management can make is calculated by comparing the outcomes when a local stressor, poor water quality, is or is not improved. We compare scenarios where poor water quality interacts additively, antagonistically or synergistically with ocean warming. There are multiple physiological mechanisms by which heat stress and poor water quality affect seagrass mortality [27]–[30]. Warming may physiologically stress seagrass by increasing respiration rates more rapidly than photosynthetic rates. Algal epiphytes may also outcompete seagrass at warmer temperatures [31]. Increased water column nitrate, as a consequence of terrestrial run-off from fertilizer use, may also promote phytoplankton and epiphyte growth and reduce light to seagrass. Eutrophication may also directly stress seagrass physiology [27].

Second, we use a community model to show that interactive effects of local and global stressors on a community of species affects species richness when the local stressor is remediated [3]. Each of these models is explained in more detail below. These examples show synergisms can accelerate declines in ecosystems, but also provide the greatest opportunity for management to benefit ecosystems. Our simple models also highlight that antagonisms can be more challenging to manage. We suggest that identifying the type and strength of interactions has not received the necessary research focus needed to support management of climate change impacts through local actions and for setting achievable management targets.

## Methods

### Population Response to Stressor Interactions: Model of Seagrass

We examined how management outcomes depended on interaction types using a model of Mediterranean seagrass density [9]. The original model was used to predict the year a seagrass bed reached a critically low density, assuming additive effects of global warming and poor water quality stress on mortality rate. We adapted this model by incorporating interactions between these stressors. It is currently not known whether synergisms or antagonisms are more likely for warming and water quality impacts on seagrass [9]. Warming and nutrient inputs may worsen declines of seagrass by simultaneously increasing growth of phytoplankton and epiphytes (multiplicative synergism) [32]. Alternatively, there may be an antagonism if nutrient inputs dominate the stress response of seagrass growth (stressor dominance, antagonism) [27]. Finally, while we are not aware of a demonstration of mitigative antagonisms for seagrass, for other aquatic primary producers poor water quality can mitigate heat stress, by reducing light stress [15].

In analyses we considered both antagonistic and synergistic interactions to demonstrate their importance for management and we hope this will stimulate further studies that will quantify the type and strength of the interaction.

We modelled declines in seagrass density using an exponential model [9]:
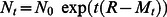
(1)where *N_t_* was seagrass density at time *t*, *R* was the recruitment rate and *M_t_* was the seagrass mortality rate at time *t*. We assumed the recruitment rate was constant over time, whereas mortality varied w*i*th temperature and a local stressor. We modified the original model for mortality rate [9] to include interactions between poor water quality (local stressor) and warming stress (global stressor):



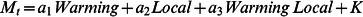
(2)This linear additive effects model is commonly used for estimating effect sizes (*a_i_*) of stresses and their interaction on ecological properties from empirical data in linear statistical models (Folt *et al.* 1999).

Adding an interaction term to Jorda *et. al*’s [9] equation increases the overall mortality rate for a synergism, or decreases the mortality rate for an antagonism. This would be unrealistic, because the overall mortality rate was estimated from field studies. Hence, for each value of *a_3_* (the interaction) we recalculated the intercept, *K*, so that the mortality rate in a base scenario was constant and consistent with field measurements (Table 1). This ensures that our scenarios of decline were consistent with those originally presented in Jorda *et al.* [9]. Including an additional interaction term in this way was not unrealistic, because the additive effects of warming and water quality stressors on mortality were estimated separately, so interactions were not considered. This was also consistent with how effect sizes are often estimated from field studies. We also explored the outcome when the interaction term was an additional source of mortality; thus, the overall mortality is higher with a synergism and lower with an antagonism. This situation would occur if the mortality rate due to individual stressors is estimated in experimental studies, but the overall mortality rate is not estimated in the field.

**Table 1:** Parameter values for the seagrass model scenarios where growth was modelled R – *M_t_* and *M_t_  =  a_1_ Warming + a_2_ Local + a_3_ Warming Local + K*.

Interaction scenario	Recruitment rate	Climate effect (*a_1_*)	Local effect (*a_2_*)	Interaction (*a_3_*)	Intercept (*K*)	Mortality rate in 2010*
Additive	0.05	0.021	0.02	0	−0.471	0.096
Synergistic	0.05	0.021	0.02	0.001	−0.497	0.096
Antagonistic – dominance	0.05	0.021	0.02	−0.0005	−0.458	0.096
Antagonistic – mitigative	0.05	0.021	0.02	−0.001	−0.445	0.096

* with both stresses.

Interaction values were chosen so that overall mortality rates were never greater than those in the model of [9]. The interaction strength (*a_3_*) is weaker for the dominance antagonism than the mitigative antagonism, to reflect the different processes that cause these types of interactions.

Mortality due to the local stressor was present in scenarios without management and absent in scenarios with management, and we used the same value as Jorda *et al.* [9] (Table 1). We increased temperature linearly at a rate of 0.38°C per decade. Jorda *et al.* [9] considered inter-annual variability in temperature and variability among climate model predictions and thus predicted mean declines with confidence intervals for seagrass. Our intent was to show the effect of interactions, rather than to quantify likely rates of seagrass decline, so we used a linear rate of temperature change to avoid unnecessary complexity. Regardless, the long-term rate of temperature change gave a similar result to the model mean in Jorda *et al.* [9]. We thus compared times to reach the 10% density threshold under different interaction types with and without management. The 10% density threshold was chosen because seagrass recovery is unlikely beyond this point [9]. We considered three classes of interaction: additive (*a_3_* = 0), synergistic (*a_3_*>0) and antagonistic (*a_3_*<0).

There is danger in extrapolating linear models so we kept our interaction strengths within bounds that gave overall mortality rates no greater than those in Jorda *et al.* [9]. We did not vary interaction strengths to be greater than 10% of the effect of temperature. This value is plausible, given interaction strengths estimated in experiments of nutrient and warming stress on seagrass [27].

The study that this model was based on has a number of inherent assumptions and limitations. In particular the methodology used to obtain the estimates of seagrass mortality rates in Jorda *et al.* [9] has been criticised [33], but see [34]. As such, the base mortality rate and the temperature effect on mortality used by Jorda *et al.* [9] may have been under or over-estimated and subsequently, the predicted year in when seagrass reaches a critically low density may be inaccurate. Further, Jorda *et al.* [9] were not able to estimate the impact of local stressors on seagrass directly using empirical measurements. To account for these criticisms, additional analyses were conducted where the base mortality rate, the temperature dependent mortality rate, the recruitment rate, and the mortality caused by the local stressor were varied. Our intent was to show qualitatively how interactions affect the outcome of management, rather than provide quantitative estimates of the year seagrass reached a critically low density. Thus, when interpreting the results from the additional analyses, we focussed on whether our qualitative results are changed, rather than quantitative differences in the year seagrass reached a critically low density.

### Community Response to Stressor Interactions: Modelling Co-tolerance

**Figure 1:**
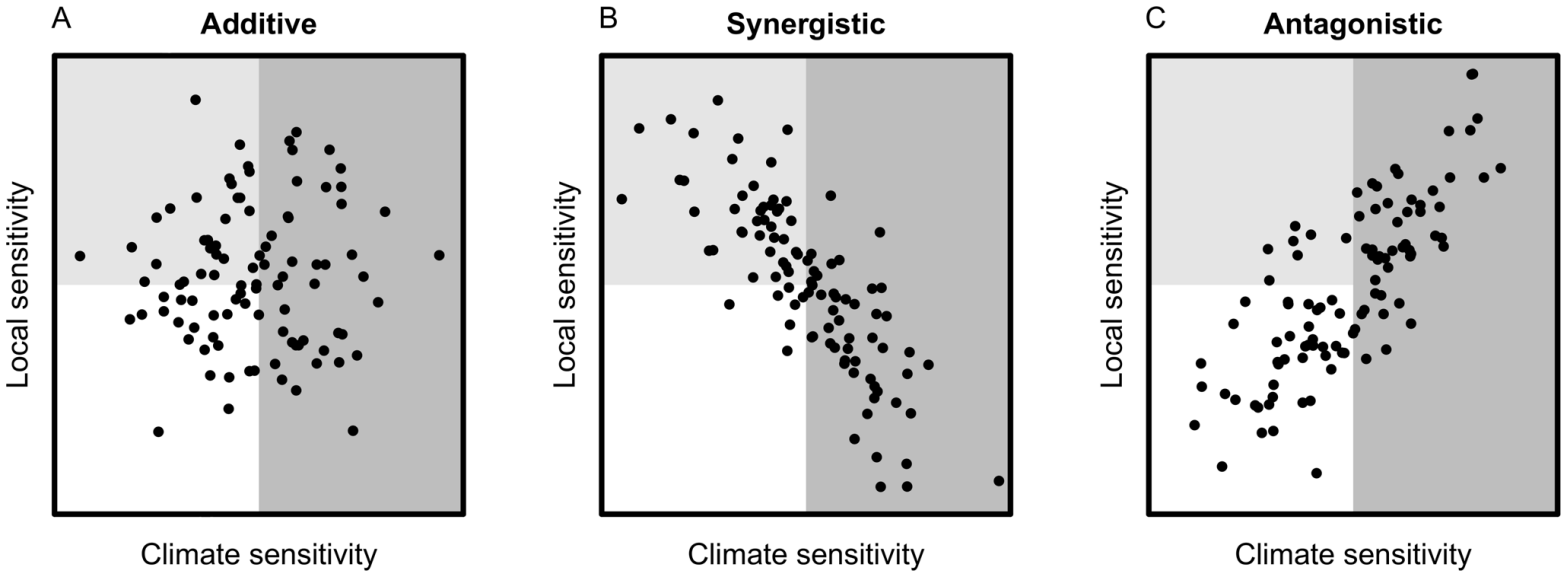
Co-tolerance of species to both climate and local stressors for three types of interactions. Species tolerances were generated randomly (nominal scales) for an additive interaction (random co-tolerance, ρ = 0), a synergistic interaction (negative co-tolerance, ρ = −0.8), and an antagonistic interaction (positive co-tolerance, ρ = 0.8). Each point represents the tolerances of a single species to the two stressors. Species in the dark grey region will be threatened by climate change stress, the local stressor will additionally affect species in the light grey region. Species in the white region will be unaffected by either stressor. The most species will be lost with a synergism and the least with an antagonism [3].

First we explain a conceptual model for community responses to multiple stressors and then below we explain how to modify the model to examine the impacts of interactions on the outcomes of management. We used the species co-tolerance model (Fig. 1, [3]). In this model, the interactive effect of two stressors on an aggregate community property, such as species richness, depends on the co-tolerance of each species to the stressors. Stressors can be pulse disturbances operating over a short time, in which case the model predicts the short-term community response. Stressors can also be press disturbances operating over a long time, or a series of pulse disturbances, in which case the model predicts the long-term community response. A stressor of a certain magnitude is assumed to remove all sensitive species. In the co-tolerance model, additional stresses only affect the community aggregate property if they affect species not sensitive to the first stress. For instance, consider an assemblage of coral reef fish species. Global warming, coral bleaching and subsequent loss of habitat over the long-term may cause local extinction of coral dependent fish species and a lower fish species richness. In this conceptual model, an additional stress, fishing, will only further reduce species richness if the remaining species are sensitive to fishing [12].

An additive effect occurs if individual species tolerances to two stressors are randomly distributed with respect to each other (Fig. 1). In this instance, habitat loss from global warming that affects 50% of the fish species will reduce species richness by 50%. Fishing pressure that also affects 50% of fish species will consequently reduce species richness by only 25%, because the 25% of species that are highly sensitive to both stressors have already been affected (a stressor dominance effect). In communities, synergistic and antagonistic interactions occur if co-tolerances are negatively or positively correlated respectively (Fig. 1). If there is strong negative co-tolerance, two stresses that each affect 50% of species will together affect close to all species, because species not sensitive to habitat loss will likely be sensitive to fishing. Whereas, if there is positive co-tolerance, species that are sensitive to habitat loss are also sensitive to fishing, so cumulative stressors will have only small further effects on the community.

We modelled community responses to multiple stressors by representing species’ co-tolerances as a multivariate normal distribution. Thus, species’ marginal tolerances are normally distributed with respect to each stressor and their co-tolerance was described by the correlation between stressor tolerances, ρ. For an extreme synergism, ρ* = *−*1*, whereas ρ* = 0* for an additive interaction and ρ* = 1* for an extreme antagonism. Increases in species richness from management were calculated by comparing the number of species affected by either or both of the local and global stressors, compared to the number of species affected by only the global stressor (see Appendix S1). Using this analysis, for the coral reef fish assemblage, we might ask how much greater species richness will be on reefs threatened by bleaching if management protects reefs from fishing by placing them in marine reserves?

Other distribution forms could also be used, but we chose the multivariate normal because it is a realistic way to simulate species tolerances to environmental factors. We further assumed that species tolerances to stressors are fixed. Stress tolerance could also be dynamic [3], however, we do not include this additional complexity, because we have illustrated dynamic responses in the seagrass case study.

In reality, co-tolerance patterns may deviate from a linear bivariate relationship. A negative convex co-tolerance curve between a local stressor (fishing) and climate change was recently described for a coral reef fish assemblage [12]. In this study, Graham *et al.* [12] assessed fish species vulnerability to population declines caused by climate and fishing. They used scientific theory and empirical assessments to assign vulnerability scores for each stressor to each species and tested these scores against independent empirical data. We used Graham *et al.*’s [12] vulnerability scores for fish species to calculate management gains from creating marine reserves (thereby reducing the fishing stressor to zero), in the presence of the climate stressor. Species tolerances are log-normally distributed in these data, however, the results are similar to those assuming a normal distribution.

## Results

### Population Response to Stressor Interactions: Seagrass Population Mortality

**Figure 2:**
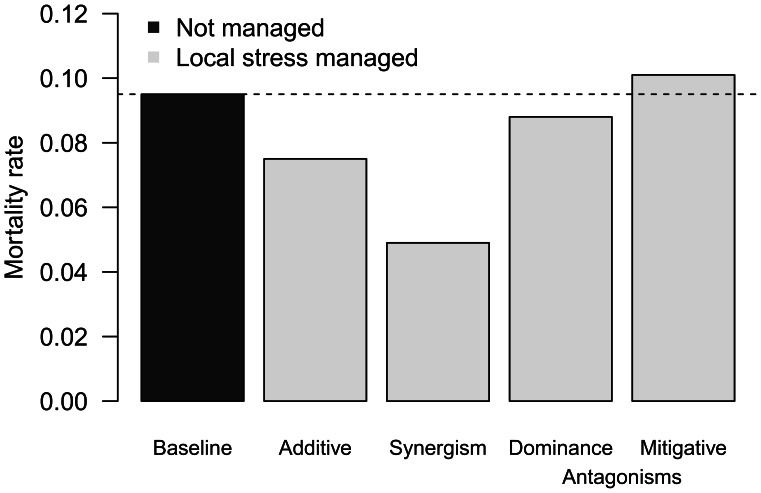
Mortality rate of seagrass for different interaction types. Mortality rate is high with both warming and the local stressor (water quality, dark grey bar). If the local stressor is improved (light grey bars), mortality rate is reduced by 0.02 per year for an additive interaction. Management with a synergistic interaction between warming and local stressor gains a greater reduction in mortality rate, whereas the reduction is small with a dominance antagonism. If there is a mitigative antagonism, mortality rate increases if the local stressor is improved.

We first used the additive effects equation with interactions (equation 2) to predict how local and global stressors affected seagrass mortality rate for different interaction types. Reducing the local stressor decreased the mortality rate when there was no interaction or a synergistic interaction (Fig. 2). Improvements were greater when the synergism was stronger (not shown). For a dominance antagonistic interaction, mortality rate decreased if management reduced the local stressor, but by a smaller amount than for an additive or synergistic interaction. By contrast, for a mitigative antagonistic interaction, mortality rate increased when the local stressor was reduced. This counter-intuitive result occurs because the antagonistic effect benefitted the ecosystem by a greater amount than the direct additive impacts.

**Figure 3:**
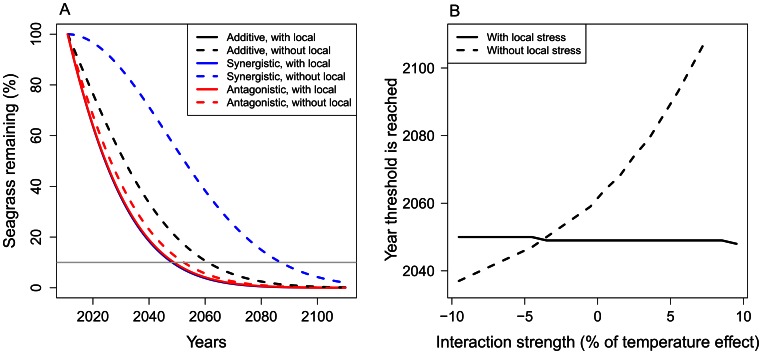
Seagrass density for different interactions with and without management of the local stressor. (A) Decline in seagrass density with and without improving the local stressor (water quality) for additive, synergistic (5% of temperature effect size), and antagonistic interactions (−2.5% of temperature effect size). The grey line represents the 10% seagrass density threshold where seagrass loss is believed to be irreversible [9]. The interaction scenarios with the local stressor almost perfectly overlay each other. (B) Year of seagrass loss for a range of interaction strengths (positive is synergistic, negative is antagonistic) when water quality is not managed (solid line) and when water quality is improved (dashed line).

Next we simulated seagrass density using the various scenarios for mortality rate. Declines in seagrass density with poor water quality were almost identical for three scenarios of interaction types without management, because we adjusted the base rate mortality to compensate for interaction effects (Fig. 3a). As in Jorda *et al.* [9], the 10% density threshold was reached in the year 2049, except for stronger antagonisms where the decline was slightly slower and the threshold is reached in 2050. When the interaction was additive, improving water quality yielded a gain of 13 years (2049 to 2062) and when the interaction was synergistic, management yielded a gain of 38 years (2049 to 2087). When the interaction was a mitigative antagonism, the density threshold was reached four years earlier if the local stressor is reduced (2050 to 2046). When the interaction was a weaker dominance antagonism, the density threshold was reached only four years later when the local stressor was reduced (2049 to 2053).

There was an exponential relationship between year of loss and interaction strength when the local stressor was improved. Stronger synergistic interactions (positive values) exponentially increased the year the density threshold was reached (Fig. 3b). Antagonistic interactions that were >∼3% of the temperature effect size were mitigative, because improving water quality at this point meant the density threshold was reached earlier than without management.

**Figure 4:**
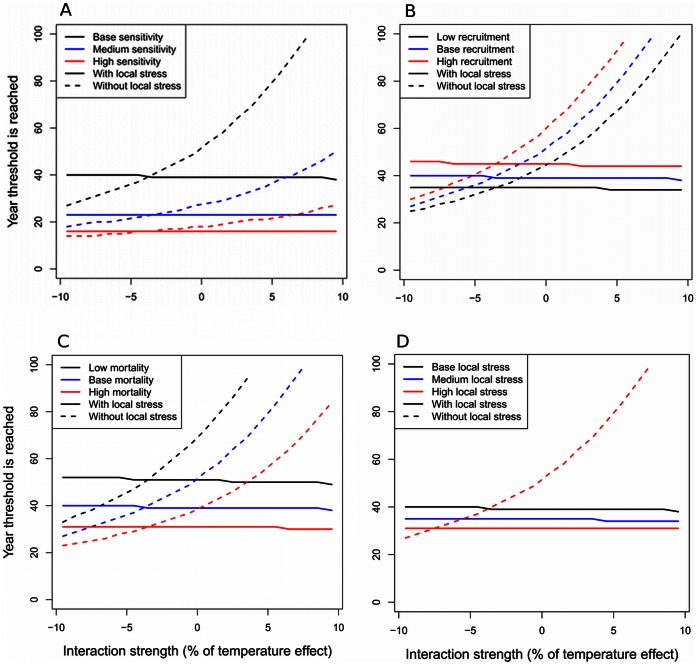
Year of seagrass loss for a range of interaction strengths, when the seagrass model parameters are varied. For each scenario, one parameter was varied while other parameters were held constant. Model scenarios when the local stressor is not managed are indicated with solid lines and scenarios when the local stressor is improved are indicated with dashed lines. Each colour indicates a different parameter value. (A) Varying warming effect sizes (parameter *a_1_*, black *a_1_* = 0.021, blue *a_1_* = 0.023, red *a_1_* = 0.025). (B) Varying recruitment rates (parameter *R*, black *R* = 0.04, blue *R* = 0.05, red *R* = 0.06). (C) Varying base mortality rates (Mortality in year 2010, *M_2010_*, black *M_2010_* = 0.076, blue *M_2010_* = 0.096, red *M_2010_* = 0.116). (D) Varying the effect of the local stressor on mortality rate (parameter *a_2_*, black *a_2_* = 0.02, blue *a_2_* = 0.03, red *a_1_* = 0.04). In (D), the simulations without the local stressor overlay each other.

The general result, that synergistic interactions gave greater gains from managing the local stressor, was robust to alternative parameter combinations. Larger temperature effect sizes reduced the benefits of improving the local stressor (Fig. 4a). Increasing the recruitment rate led to greater gains for management when there was a synergism and greater losses when there was a mitigative antagonism (Fig. 4b). Decreasing the initial mortality rate had a similar effect to increasing the recruitment rate (Fig. 4c). Increasing the mortality caused by the local stressor meant the density threshold was reached earlier (Fig. 4d). This also improved the gain from management, because by removing the local stressor management affected a greater fraction of the overall mortality rate.

**Figure 5:**
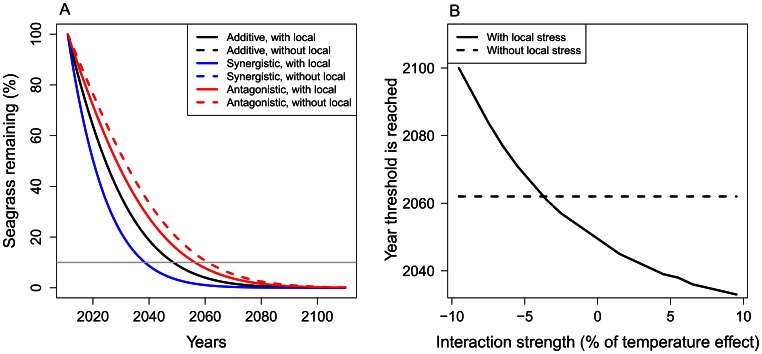
Seagrass density declines for different interaction types when mortality from the interaction term is additional to the base rate mortality. (A) Seagrass density decline with and without the local stressor for additive, synergistic (5% of temperature effect size) and antagonistic interactions (−2.5% of temperature effect size). The grey line represents the 10% seagrass density threshold where seagrass loss is believed to be irreversible. The interaction scenarios with the local stressor almost perfectly overlay each other. (B) Year of seagrass loss for different interaction strengths (positive is synergistic, negative is antagonistic) when the local stressor is not managed (solid line) and when the local stressor is removed (dashed line).

We next considered a case where the interaction is an additional source of mortality, so that overall mortality was higher with a synergism and lower with an antagonism. In this case, the rate of decline with both stressors was greater with a synergism and slower with an antagonism (Fig. 5a). Whereas, if management reduces the local stressor, the outcome was the same regardless of the interaction. The year the threshold was reached was constant amongst interaction types when the local stressor was mitigated (Fig. 5b). Whereas the year of loss occurs exponentially earlier for increasingly synergistic interactions when the local stressor was present. The general conclusion that more synergistic interactions provide greater gains still held.

### Community Response to Stressor Interactions: Stressor Co-tolerance of Coral Reef Fish

**Figure 6:**
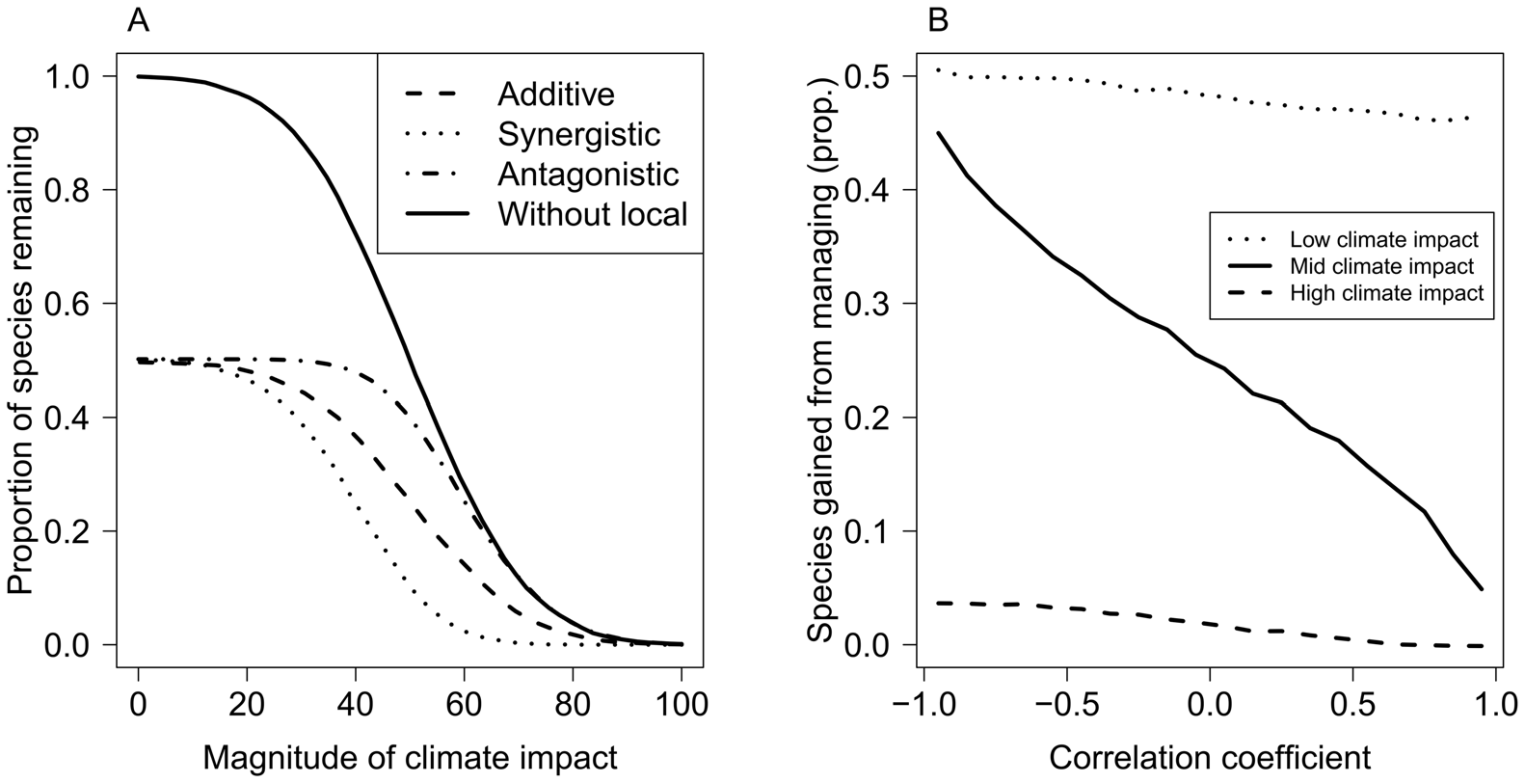
Predicting species loss with co-tolerance relationships. (A) The proportion of species remaining out of 10 000 for different magnitudes of warming temperature. Dashed lines show species remaining with local and global stressors for different interactions. The local stressor was assumed to affect half the species in the absence of the climate stressor. Increasing magnitudes of the climate stressor reduce the proportion of species remaining. The solid line shows the species remaining without the local stress (same for all interaction types). (B) Species gained by reducing the local stressor for different cotolerance strengths (x-axis is the correlation coefficient for stressor responses, negative is synergistic and positive is antagonistic). Management will have the greatest benefit at low climate impact sites (dotted line) and little benefit at high climate impact sites (dashed line), regardless of the interaction type. At moderate impact sites however, there are greatest management gains when there is negative co-tolerance.

We first considered the number of species remaining when subject to both a global stressor (e.g. habitat loss by coral bleaching) and a local stressor (e.g. fishing pressure) for different species co-tolerance patterns. Regardless of the co-tolerance type, the number of species conserved by removing the local stressor was the greatest when the magnitude of the climate stressor was the smallest (Fig. 6). If the number of species lost in response to a global stressor was a large proportion of the total number of species, then removing the local stressor had little benefit. The type of co-tolerance had the greatest effect on the number of species conserved when the global stressor was of intermediate magnitude (Fig. 6). The most species were benefitted by local management if there is a negative co-tolerance relationship (synergism), whereas the fewest species benefit if there was a positive co-tolerance relationship (antagonism). Hence, while antagonisms (positive co-tolerance) resulted in loss of fewer species, management that assumed an additive or synergistic interaction would not be effective.

**Figure 7:**
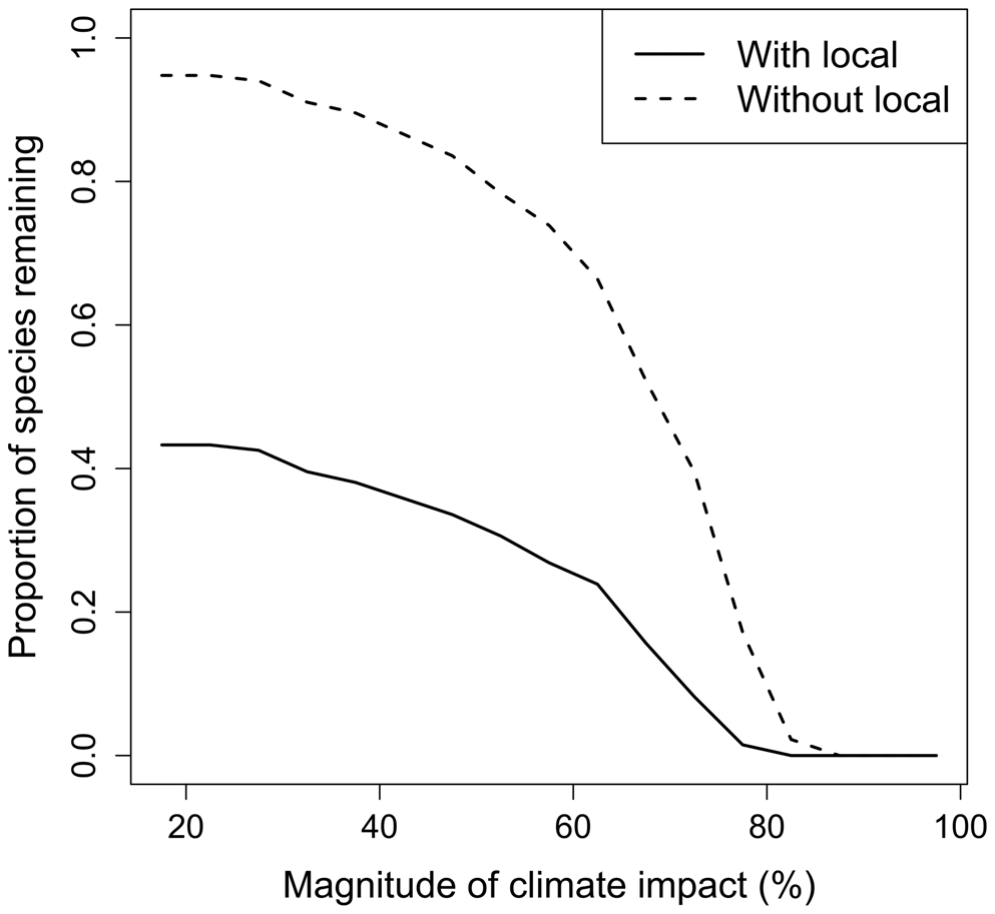
Empirical example of management effectiveness for negative co-tolerance. Proportion of coral reef fish species remaining out of the 134 observed for different magnitudes of climate change impacts (data from [12]). The solid line is for a fishing stressor affecting 50% of species and the dashed line without the fishing impact.

In reality, coral reef fish may show negative co-tolerance to climate and fishing, with convex rather than linear co-tolerance. Modelling the response of coral reef species richness to reductions in fishing indicated that large gains in species richness were made for intermediate climate impacts (Fig. 7). Thus, this empirical example with a convex rather than linear relationship, and with species distributed log-normally on the stressor axes, was consistent with the theoretical model of species co-tolerance (Fig. 7).

## Discussion

We explored the impact of managing local stressors when there are interactions with global stressors using two kinds of ecosystem model. Both models demonstrated that management to reduce a local stressor has the largest benefits for ecosystems when there are synergistic interactions. By contrast, reducing the local stressor gave smaller benefits when there was an antagonism, or could even worsen stressor impacts, if there was a mitigative antagonism. Therefore, knowing the type of interaction is important to determine the expected benefits of reducing a local stressor.

There was little that local management could do to counter severe impacts of climate change on populations and communities, even with synergistic interactions or negative co-tolerance relationships. In these cases, reduction of global greenhouse gas emissions is necessary to slow degradation of ecosystems. Without mitigation of global warming, seagrass populations in the Mediterranean Sea will likely fall below the 10% density threshold within the next 100 years regardless of local management interventions [9] (but see [33]) or the type of interaction between local and global stressors. However, management of local stresses did buy more time when there was a synergistic interaction. This added time may provide an opportunity for evolutionary adaptation, development of alternative local management actions, and for mitigation of global warming. Further, climate impacts are spatially variable so there may be opportunities to identify refuges where local management can have the greatest benefits [26].

The co-tolerance model implies that greater numbers of species will be benefitted by local actions when there are negative co-tolerance relationships (synergisms). Whether these species are important for community assembly and ecological functions is an important next question. For instance, different functional groups of coral reef fish species may fall at different places on the co-tolerance curve [12]. This has important implications for ecosystem function because different groups of coral reef fish perform important functional roles on reefs, such as herbivory [22], [35]. For coral reef fish, functionally important species tend to have greater sensitivity to fishing than to climate, so management of fishing can contribute to conserving these functions [12]. It may be the case in other systems that functionally important species are more sensitive to climate than the local stressor. In these systems, conservation should focus on reducing stressors in refuges from climate change impacts.

### Measuring Interactions

Estimates of interaction effect sizes and types between local and global stressors are helpful for identifying appropriate management actions to global impacts on ecosystems. A major caveat to our models was that appropriate estimates of interaction effect sizes were not readily available. Manipulative studies in the field and laboratory can empirically estimate interaction effect sizes. Concurrent time-series data of ecosystem indicators and stressor values can also be used to estimate interactions from monitoring data. To be most informative for management, studies of multiple stressors could consider interactions between local and global stressors, rather than solely between global stressors [36], [37].

While it has not been widely applied to date, the co-tolerance model provides a complementary method for predicting interaction effects on community traits. For instance, Graham *et al.* [12] characterised the climate and fishing sensitivity of a coral reef fish community as a negative co-tolerance (synergistic) relationship. This conceptual model is still in early stages of development and field tests of how well it predicts community responses to multiple stressors are needed (e.g. [12]). Importantly, immediate stress responses and recovery dynamics of species may lead to very different communities. Species’ environmental responses may better be characterised on three or more axes, which encompass groups of species that are numerically dominant in stable productive environments, stressful environments and post-disturbance [38], [39]. The community response to multiple stressors will therefore depend on both the intensity and frequency of disturbances and the variability in recovery rates of species.

A challenge for predicting responses to management of a local stressor is that stressors indirectly affect state variables of management interest, through their direct effects on physiological and population processes, such as mortality rates. For the seagrass population model, warming and water quality stressors had linear effects on mortality rate, but because mortality is cumulative, the stresses had non-linear effects on seagrass density. Making empirical measurements of processes is more time consuming than measurements of state and often not practical in experimental settings. For instance, estimating mortality rate requires at least two counts of population size for each stressor’s level [29], and it is difficult to obtain unbiased estimates of mortality in many aquatic organisms, such as seagrass [33], [40]. It is important to estimate mortality rate accurately for predicting declines and recovery in populations. However, our qualitative findings from the population model were robust to higher and lower mortality rate estimates.

Stress responses of populations and communities should not always be expected to be as straightforward as in the simple models used here. Studies of seagrass responses to warming and eutrophication indicate that stress responses can involve multiple traits, including changes to photosynthesis and respiration rates and interactive effects can vary for these different physiological traits [27]. Further, the physiological response to an environmental stressor can be non-linear, so extrapolation from linear models may fail to predict the ecological response to management. If the physiological response is a monotonic function of the stresses, our general result, that managing under a synergism gives greater benefits than under an antagonism, will hold for population density. However, physiological responses to stressors can also be non-monotonic. For instance, growth, mortality and reproductive rates for a species increase from cool to moderate temperatures, but rapidly decline beyond an optimal temperature [41]. Whether seagrass responds to changes in an environmental variable as a stress or a benefit may also vary seasonally [30]. For community traits, species may shift their tolerance of one stressor in response to presence of another stressor, meaning that co-tolerance curves can be dynamic [3]. For instance, fished species may be more sensitive to climate change impacts than unfished species [42].

Investigating non-linear and multiple trait stress responses in manipulative studies requires large amounts of replication and often logistically unfeasible experimental designs. For practical reasons, most experimental studies of interactions use only two levels for each stressor [16], [17], [37]. Further, there are often scale disparities in stressor impacts that make empirical estimation of interactions challenging. For instance, the effects of ocean acidification on physiology of marine organisms are amenable to laboratory experiments, whereas the effects of fishing on food webs are not. Conducting adequately replicated manipulative experiments in field sites may be prohibitively expensive and often, politically unacceptable [43].

### Models to Inform Management

There are several models that could be used to inform appropriate management actions when there are interactions between local and global stressors. Stressor co-tolerance relationships can be built on the basis of literature reviews and may be a rapid way of estimating interactions. Analyses such as Graham *et al.*’s [12] can inform management directly, such as in predicting how placing marine reserves will affect reef communities impacted by warming. Measurements of stressor interactions on multiple physiological traits, such as seagrass photosynthesis and respiration, can be integrated into process-based models to extrapolate outcomes for ecological states under different management responses (e.g. [44]). Process-based models can also integrate stressor impacts from experimental studies and larger-scale field studies, such as effects of fishing and ocean acidification on marine food webs [45], [46]. Considering interactions between species may be particularly important, because species interactions may often be a mechanism for mitigative effects. This is a caveat to the co-tolerance model, which currently does not consider dynamic interactions.

### Implications and Conclusions

Antagonisms are often perceived as less of a concern than synergisms, because impacts of multiple stressors on ecosystems will be smaller, so management in these circumstances may be viewed as less urgent. This view has contributed to the bias in the present literature towards analyses of synergisms [16]. Our analysis demonstrates a need for a more balanced research agenda that identifies both synergisms and antagonisms. Reducing stressors that interact antagonistically may be a lower priority for management, but if an antagonism is assumed to be additive or synergistic, attempts at improving ecosystems may be foiled. Acting when there are antagonisms is unlikely to have negative effects on the ecosystem, but it does waste effort and resources that could be used to have greater benefits elsewhere. For instance, global warming and fishing may have an antagonistic effect on coral reefs, so marine reserves will be of greatest benefit to corals if they are placed in refuges from warming [26].

Resources to determine the type of interaction may not be available when a decision regarding management of a local stressor must be made, so management must assume that a particular type of interaction is prevalent. Previous research has suggested it is conservative to assume additive interactions (e.g. [1], [47]). Assuming that additive interactions are common is an appropriate middle ground if the aim is to estimate the potential impact of stressors on an ecosystem and it is not known whether synergistic or antagonistic interactions are more likely. Our research indicates that for making management decisions, it can be more conservative to assume either antagonistic or synergistic interactions, depending on the goals of management [23] and the action being taken to address a local stressor. For instance, the choice may be the location at which to act on a local stressor (such as where to place marine reserves to reduce fishing pressure) and the goal to ensure some sites maintain healthy ecosystems. In this instance, the most conservative strategy is to assume an antagonistic interaction, and undertake remediation of local stressors in climate refuges. Reducing local stressors in sites impacted strongly by climate change will provide no benefit if there is an antagonism. Alternatively, the choice may be to act, or not act, to reduce a local stressor in one location. In this case, assuming an additive or synergistic interaction is more conservative from an environmental perspective, because it implies taking action rather than no action. Further research, using risk analysis techniques, is needed to elucidate what the most conservative assumption is for different management contexts when interactions cannot be estimated.

Mitigative antagonisms may be particularly challenging for management, because outcomes may ultimately be worse than under no management. In particular, interactions need to be considered across food webs, rather than just individual species, because mitigative antagonisms commonly show up in food web models [45]. Future syntheses are needed that determine the prevalence of mitigative antagonisms, so the magnitude of this management risk can be identified.

The challenges antagonisms pose for ecosystem management have previously been pointed out by other authors, where it has been argued that management of ecosystems with antagonisms requires action on both global and local stresses [48]. With delays on mitigation of global stressors, management requires alternative approaches that can work at local scales. This study suggests management priorities can be adapted to accommodate interactions with climate change, provided climate impacts are not severe. For instance, local stresses that interact synergistically, rather than antagonistically, with climate change should be a priority for management action. Climate change impacts are also spatially variable, so management faced with antagonisms could identify refuges from climate change where management of local stresses will have greatest benefits (e.g. [23]). Incorporating these interactions into schemes for prioritising management action (e.g. [18], [19], [49] is an important next step.

## Supporting Information

Appendix S1 (DOCX)
