# Supplementary material for: Managing for Interactions between Local and Global Stressors of Ecosystems
Source: PLoS One. 2013 Jun 12;8(6):e65765. doi: 10.1371/journal.pone.0065765 (PMC3680442; doi:10.1371/journal.pone.0065765)
Supplement: Appendix S1 — (DOCX) [file pone.0065765.s001.docx]

**Supplementary Information:**

**Managing for interactions between local and global stresses of ecosystems**

Christopher J. Brown^*^

The Global Change Institute and the School of Biological Sciences, The University of Queensland, St Lucia, Queensland, Australia

Phone: +61 7

christo.j.brown@gmail.com

*To whom correspondence should be addressed

Megan I. Saunders

The Global Change Institute, The University of Queensland, St Lucia, Queensland, Australia

Hugh P. Possingham

School of Biological Sciences, The University of Queensland, St Lucia, Queensland, Australia

Anthony J. Richardson

Climate Adaptation Flagship, Commonwealth Scientific and Industrial Research Organisation, Marine and Atmospheric Research, Ecosciences Precinct, Dutton Park, Queensland, Australia.

Centre for Applications in Natural Resource Mathematics, School of Mathematics and Physics, The University of Queensland, St Lucia Queensland, Australia.

**Appendix SI1: Equations for stress co-tolerance**

For an additive interaction, the proportion of species that are affected by at least one of two stressors, *S_1_* and *S_2_*, is described:

*P(S_1_ U S_2_) = P(S_1_) + P(S_2_) – P(S_1_)*P(S_2_)*

Synergistic and antagonistic interactions occur if co-tolerances are negatively or positively correlated respectively (Fig. 1). Thus, the proportion of species affected depends on the co-tolerance:

*P(S_1_ U S_2_) = P(S_1_) + P(S_2_) – P(S_1_)*P(S_2_ | S_1_)*

Where *P(S_1_)*P(S_2_ | S_1_)* describes the co-tolerance. For extreme negative co-tolerance, the correlation between species will be -1, so:

*P(S_1_ U S_2_) = P(S_1_) + P(S_2_)*

For extreme positive co-tolerance, the correlation will be 1, so:

*P(S_1_ U S_2_) = max( P(S_1_), P(S_2_) )*
